# An unusual etiology of bilateral pulmonary nodules: Another challenge of hide and seek

**DOI:** 10.1016/j.rmcr.2021.101482

**Published:** 2021-07-27

**Authors:** Anam M. Elarabi, Mousa Hussein, Aisha Aladab

**Affiliations:** Pulmonary Department, Hamad General Hospital, Hamad Medical Corporation, Doha, Qatar

**Keywords:** Extraskeletal chondrosarcoma, Pulmonary metastases, Penile tumor

## Abstract

An elderly gentleman presented with bilateral pulmonary nodules found incidentally during the workup of acute pancreatitis. He did not have any respiratory or urogenital symptoms. A biopsy of the lung nodule revealed myxoid mesenchymal neoplasm of extraosseous origin. As the patient was asymptomatic, only the increased uptake in the penile shaft base on the whole-body PET-CT study yielded a diagnosis of primary penile chondrosarcoma after biopsy. A rare presentation of chondrosarcoma in an elderly gentleman highlighting the importance of obtaining a histopathological specimen, as the prognosis of metastatic diseases is widely variable.

## 1 Introduction

Chondrosarcomas (CHS) are a common primary bone tumor third only to myeloma and osteosarcoma. They originate from the hyaline cartilage differentiation and commonly affect middle age groups of equal gender distribution patients [1]. 10–15 % of them are considered high-grade with a higher tendency to metastasize and carry a worse prognosis [2]. Tumors originating at the axial skeleton and pelvis are usually hyaline cartilage matrix abundant with characteristically moderate cellularity; they fall under the “chondrosarcoma grade 1″ category, and they seldom metastasize [3]. Chondrosarcomas most commonly originates in the pelvis and can sometimes arise in soft tissues in proximity to the bones [4,5]. Apart from a recently described case in a young male, penile chondrosarcoma is a rare entity in clinical practice and has not been previously reported in the elderly population [5]. Our patient presented with a bilateral variable in size pulmonary nodules, which were later found to be metastasis from penile chondrosarcoma.

## 2 Case presentation

**Fig. 1:**
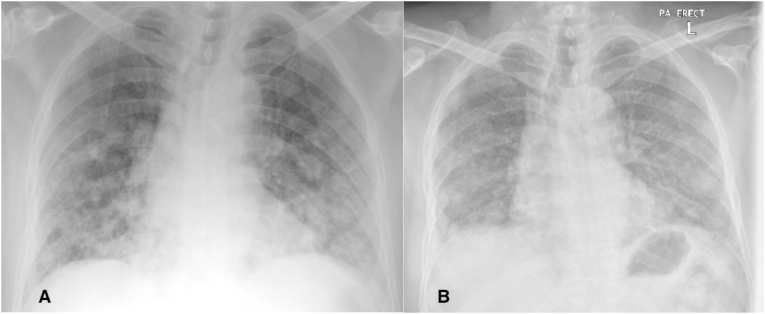
(A and B): Chest X-Ray showing bilateral pulmonary nodules. (A) The new film upon presentation. (B) 2-years before.

**Fig. 2:**
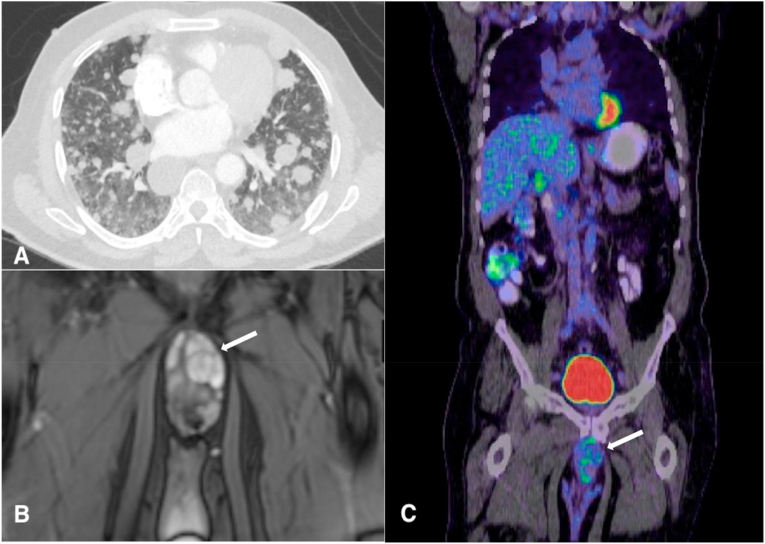
(A, B, and C): (2 A) Computerized Tomographic chest film confirming bilateral rounded pulmonary nodules. (2 B) MRI pelvis showing the penile shaft basal tumor (Highlighted by the white arrow). (2 C) Coronal PET-CT confirming the penile uptake (Highlighted by the white arrow).

An 80-year-old man has presented with acute pancreatitis secondary to biliary stones. He did not have any shortness of breath, cough, or chest pain; he also denied fever and change in appetite or weight. Physical examination was remarkable for abdominal tenderness, which has resolved gradually in response to the supportive management. Except for raised Pancreatic enzymes, his laboratory investigations were unremarkable (Table 1). Chest X-Ray showed bilateral multiple rounded balloon-like opacities, a finding described two years ago on chest x-ray film but was not investigated (Fig. 1A and B). A CT scan chest with contrast confirmed the findings of bilateral wide-spread rounded lung lesions of 5–30 mm in diameter (Fig. 2A).

**Table 1:** Relevant lab investigations.

Investigation	Result	Normal range
WBC count	6.9	4–10 × 10^3/uL
Platelet count	184	15–400 × 10^3/uL
Hgb	13	13-17 gm/dL
Creatinine	78	62-106 μmol/L
C- Reactive protein	79	0–5 mg/L
Alanine aminotransferase	302	0–41 U/L
Direct bilirubin	37.2	0–5.1 mmol/L
Pancreatic Amylase	1439	13–16 U/L
Pancreatic Lipase	3000	13–16 U/L
Lactate dehydrogenase	271	135–225 U/L
CA 19-9	21.9	0–29 U/mL
CEA	0.9	3.8–5.0 μg/L

**Fig. 3:**
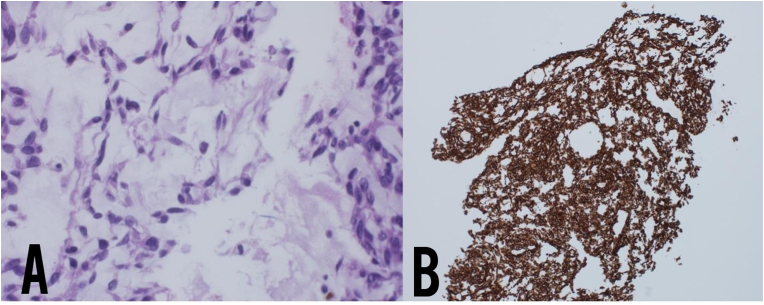
Lung nodule biopsy showing tumoral proliferation of spindle to stellate-shaped cells forming anastomosing cords and lobules within the myxoid stroma. Vimentin is diffusely positive, and p63 shows patchy positivity. S100 shows focal positivity.

The lung nodule's CT-guided biopsy confirmed a myxoid mesenchymal neoplasm and showing tumoral proliferation of spindle to stellate-shaped cells forming anastomosing cords and lobules within the myxoid stroma (Fig. 3A). Vimentin is diffusely positive, and p63 shows patchy positivity with EWSR1 rearrangement on Fluorescence in Situ Hybridization (FISH) study. (Fig. 3B). PET-CT demonstrated mild FDG uptake in the pulmonary nodules and the pelvic region (Fig. 2C). MRI pelvis revealed an infiltrative penile shaft base lesion involving the corpora spongiosum (Fig. 2B), a biopsy that showed myxoid sarcoma compatible with extraskeletal chondrosarcoma. We started the patient on Pazopanib and achieved remission for the following year.

## 3 Discussion

Pulmonary metastases are seen in 20–54 % of extrathoracic malignancies and are the second most frequent site of metastases from other organs [6]. The hematogenous route is the most common way of metastasis, particularly in cancers of the head and neck, thyroid, adrenals, kidneys, and testes, and malignant melanoma, soft-tissue sarcomas, and osteosarcoma [7]. Despite the advancement in isolated lung metastasis treatment, multiple pulmonary metastases usually reflect a poorer prognosis and higher mortality rates [6].

Not exclusively malignant, bilateral pulmonary nodules can be from benign etiologies like granulomas, chondromas, hamartomas, intrapulmonary lymph nodes, fibrosis, and inflammatory nodules [8].

Penile tumors are commonly either metastatic disease or squamous cell carcinomas; however, after an extensive review of the literature, one young patient with primary high-grade penile chondrosarcoma was reported by Alhubaishy et al. after presenting with a painless mass [5].

Herein we report a patient with primary penile chondrosarcoma diagnosed after presenting with incidental finding of multiple lung metastases in the elderly population. A finding that ideally triggers a series of radiological and histopathological workups essential to diagnose, identify the primary source, and subsequently plan the treatment.

The presence of an old radiograph with similar findings reassured the medical team about the tumor's slow-growing nature. A whole-body PET CT scan was deemed necessary to identify the metastases' source after the nodule's biopsy results showing myxoid mesenchymal neoplasm. After the MRI pelvis confirming the penile shaft base tumor, a biopsy revealed myxoid sarcoma compatible with extraskeletal chondrosarcoma.

Data are scarce about the best diagnostic and treatment approach for such patients, given its rarity. According to the European Reference Network for rare adult solid cancers (EUROCAN), all pelvic chondrosarcomas should be treated by excision with wide margins followed by chemotherapy, particularly those who are dedifferentiated and high-grade [9]. After multidisciplinary team discussion, we offered the patient Pazopanib, a tyrosine kinase inhibitor, 800 mg daily to date. He remained asymptomatic while on treatment, and a follow-up PET-CT confirmed stable lung nodules and partial metabolic response at the penile base.

## 4 Conclusions

A high index of suspicion should always be maintained during the investigation of multiple pulmonary nodules. Patients sometimes have an atypical presentation with incidental findings during the workup of other clinical conditions. In Our patient, the biopsy was a game-changer as their results led to a more comprehensive workup approach and, eventually, favorable results despite the patient's advanced age. This is the first case of penile extraskeletal chondrosarcoma presenting with metastatic slow-growing pulmonary nodules.

## Declaration of competing interest

The authors declare that they have no known competing financial interests or personal relationships that could have appeared to influence the work reported in this paper.
